# The Development, Internal and External Validation of a Circumcision Complications Risk Calculator for an African Population: Prevention of Circumcision Complications via Pre-circumcision Complication Risk Profiling in Ghana

**DOI:** 10.7759/cureus.86716

**Published:** 2025-06-25

**Authors:** Frank Obeng, Sylvester A Boakye, Banabas Kpankyaano, Daniel S Seshie, Jephtha Owusu Boateng, Evans K Zikpi, Eric N Okai, Wofa B Appiateng, Obed K Amenyo, Justice Dzomeku

**Affiliations:** 1 Department of Surgery, University of Health and Allied Sciences, Ho, GHA; 2 School of Medicine, University of Health and Allied Sciences, Ho, GHA; 3 Operative Room - Nursing, Ho Teaching Hospital, Ho, GHA; 4 Ho Polyclinic, Ghana Health Service, Ho, GHA

**Keywords:** circumcision in males, ghana, logistic regression models, prevention and control of circumcision complications, risk assessment, sociodemographic characteristics as digital biomarkers, validation study, web/mobile applications

## Abstract

Background and objective

Circumcision complications from clinical and non-clinical procedures pose significant health risks in Ghana. In light of this, tools that predict and prevent these mishaps using individual sociodemographic risk factors as digital biomarkers are urgently needed. Mobile health (mHealth) technologies offer a promising platform for improving circumcision safety through digital risk profiling. In this study, we aimed to develop a mobile app-based digital risk calculator for preventing circumcision complications in Ghana by leveraging digital biomarkers and risk profiling.

Methods

We conducted a five-year retrospective analysis of hospital-based data involving a total of 217 participants (186 for model development and 31 for external validation), identifying key risk factors including demographics, circumciser skill level, and provider facility type. Embedded, but not explicit, was the circumcision-seeking behavior of participants and thus, the geospatial distribution of complications. These variables were integrated into a logistic regression model. Internal and external validation of the model was conducted. The model was then deployed via an "R: A Language and Environment for Statistical Computing" platform, embedded into a mobile app designed for healthcare providers and parents. Pilot testing assessed app usability in 30 adult participants.

Results

The app categorized patients into low, moderate, and high-risk groups. The diagnostic model achieved a specificity of 96.08%, a positive predictive value (PPV) of 64.71%, and a negative predictive value (NPV) of 86.98%, correctly classifying 84.95% of cases. Sensitivity was 33.33%. The Hosmer-Lemeshow goodness-of-fit test (χ2 = 11.05, p *= 0.199*) confirmed the model fit. The receiver operating characteristic (ROC) analysis showed excellent discrimination [area under the curve (AUC) = 0.8895]. External validation and usability testing yielded favorable results.

Conclusions

This mobile app offers a valuable tool for real-time circumcision risk assessment, enhancing safety outcomes. Future research should aim to incorporate machine learning to optimize predictive performance.

## Introduction

Male circumcision is a widely practiced procedure in Ghana, undertaken for cultural, religious, and medical reasons. However, circumcision mishaps - such as severe bleeding, infection, partial or total penile amputation - remain a significant source of morbidity, especially in non-clinical settings [1-3]. Despite improvements in clinical practice, mishaps persist due to factors including unskilled circumcisers, poor procedural conditions, and lack of risk awareness [4-6]. Digital risk profiling using mobile health (mHealth) technologies has been increasingly adopted in other fields to enhance preventive care [7-9]. However, its application in circumcision risk prevention remains underexplored. We propose a mobile application to stratify circumcision risk in real-time based on individualized data, aiming to guide providers and parents towards safer circumcision decisions. This study details the development, validation, and pilot testing of such a mobile application, aligning with the STROBE (Strengthening the Reporting of Observational Studies in Epidemiology) guidelines [10].

The study aimed to develop a mobile application for predicting the risk of complications associated with male circumcision in Ghana, using clinical and demographic predictors. The objectives were as follows: to identify key sociodemographic, and provider-related risk factors associated with circumcision complications through retrospective data analysis; to analyze the circumcision-seeking behaviors of the participants based on their sociodemographic characteristics; to construct a predictive model using logistic regression techniques based on the identified risk factors; to internally and externally validate the predictive model to ensure its reliability, discrimination, and calibration across different population settings; to develop a functional and user-friendly mobile application that integrates the validated risk model for real-time circumcision complication risk profiling; and finally, to evaluate the performance, usability, and diagnostic accuracy of the mobile app through pilot testing and metrics such as sensitivity, specificity, and AUC [the area under the receiver operator characteristic (ROC) curve].

This article was previously presented as a meeting abstract at the 2025 Innovations in Healthcare Quality and Safety Summit (IHQS), held on May 17, 2025.

## Materials and methods

Conceptual framework

**Figure 1:**
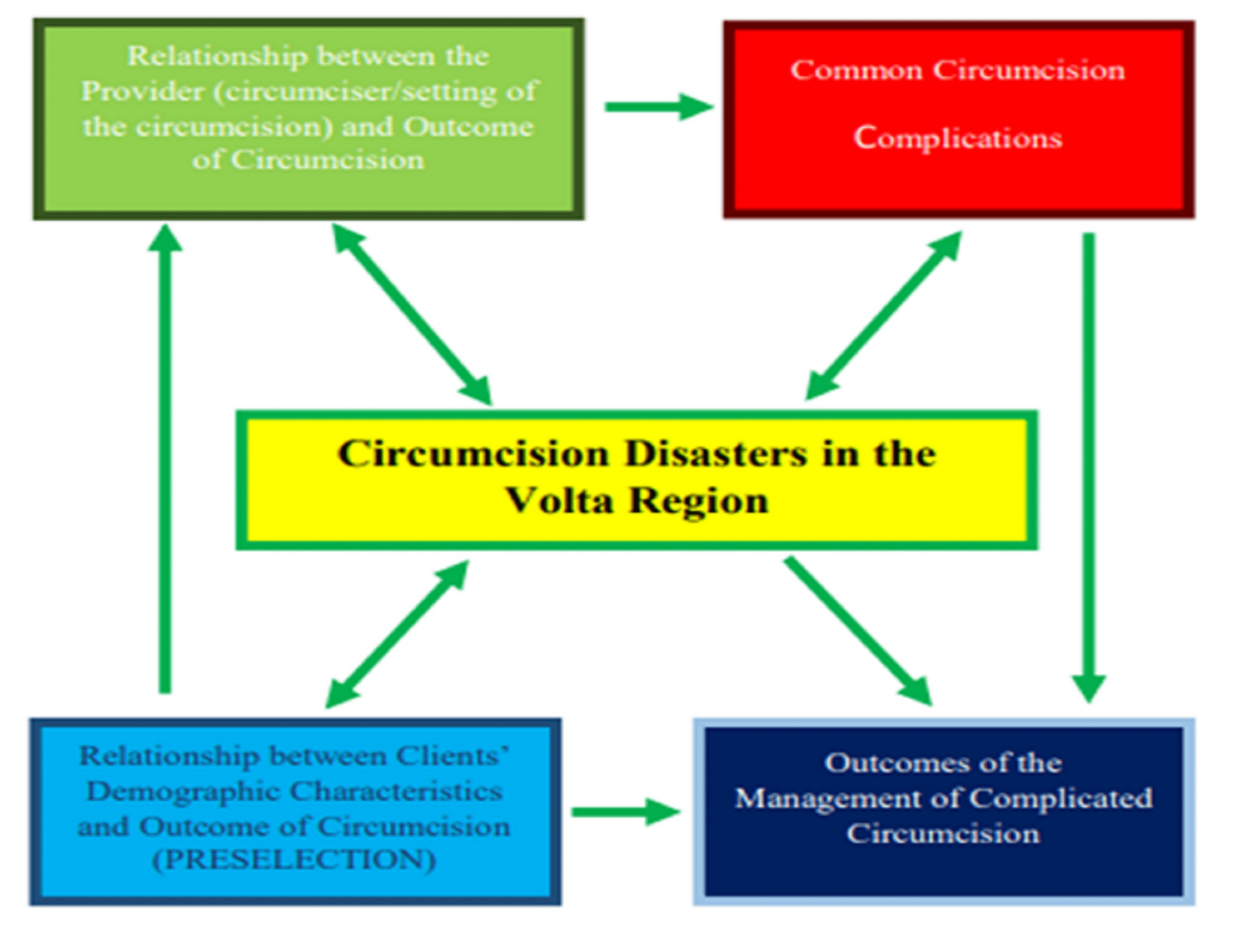
Conceptual framework diagram for the study Conceptual framework illustrating the factors influencing circumcision outcomes This diagram outlines the key variables and hypothesized relationships that affect circumcision outcomes. Factors are categorized into patient-related, provider-related, procedural, and contextual domains. Arrows indicate the direction of influence, demonstrating how these elements interact to impact the overall outcome of circumcision procedures Source: authors’ original creation

The conceptual framework for this study involves a depiction of the multidimensional contributors to circumcision disasters in the Volta Region (Figure 1), structured to inform the development of a predictive risk model and a digital application. It identifies two primary determinants influencing circumcision outcomes: provider-related factors (such as circumciser type and facility setting) and client-related demographic characteristics (such as age, parental education, and residence). Embedded, but not explicit, is the circumcision-seeking behavior of clients and thus, the geospatial distribution of complications. These inputs serve as digital biomarkers for preselection and risk profiling, aligning with the increasing global adoption of predictive health models [7,9,11].

The framework demonstrates that both provider and client characteristics independently and jointly influence the likelihood of common circumcision complications, which in turn impact the overall burden of circumcision disasters. Importantly, it also captures how these complications feed forward into the management outcomes of complicated circumcision, a crucial feedback loop for assessing the long-term utility and impact of preventive interventions (Figure 1).

This model informs the development of a risk calculator that uses structured sociodemographic and procedural inputs to assign patients into low, moderate, or high-risk categories, thereby guiding safer circumcision decisions [6,11,12,13]. The integration of this logic into a mobile application promotes real-time, point-of-care risk assessment [14,15,16], especially in underserved settings where traditional and non-clinical circumcisions are common and poorly regulated [3,4,5]. By systematizing the risk stratification process, the app facilitates targeted interventions, caregiver counseling, and policy-level preventive strategies, in line with mHealth strategies shown to be effective in other public health domains [7,16,17, 18].

Ultimately, the framework not only supports predictive modeling but also enables implementation science applications, transforming retrospective data into a proactive tool to reduce complications, enhance provider accountability, and empower clients with informed choices (Figure 1), as recommended in some surgical safety literature [1,14,18].

Study design and participants

We conducted a five-year retrospective analysis of hospital-based data from January 2019 to June 2024 at the main teaching hospital serving the southeastern parts of Ghana. This was followed by model development, validation, and usability testing. A total of 217 participants (infants and children who had undergone circumcision) were enrolled after obtaining parental consent. An additional 30 adults participated in the usability testing.

Study setting and population

Setting

This study was conducted at a tertiary hospital that serves the southeastern part of Ghana. It caters to individuals who opt for in-hospital circumcision in-house and those who are referred due to complicated circumcisions, from satellite facilities.

Participants

We included all patients who presented with circumcision requests or who were referred from other satellite facilities with circumcision complications.

Variables

Key variables extracted included demographics (age, parental education, parental occupation, residence, religion, ethnicity). Clinical variables included the skill level of the circumciser and the facility offering the circumcision.

Inclusion and exclusion criteria

Participants were eligible if they were male and were scheduled to undergo circumcision during the study period. Exclusion criteria included pre-existing urogenital anomalies, incomplete data, or parental refusal to consent.

Data sources/measurement

Data were collected retrospectively from multiple sources, including emergency department records, urology ward admission logs, operating theater registers, procedure books (for both major and minor surgeries), and discharge summaries. Also included were the pediatric wards and child welfare clinics. Trained clinical research assistants used a standardized data abstraction form designed by the research team to ensure consistency. Ambiguous cases were discussed with the lead investigators for resolution.

Bias

To reduce selection and misclassification bias, all records within the defined period and criteria were exhaustively reviewed. A uniform data extraction template was used. Independent reviewers performed cross-checking to validate the accuracy of the extraction.

Data collection tool

A standardized Microsoft Excel spreadsheet developed for the study, to ensure consistency and accuracy, was used for data collection. Using the structured questionnaire, we collected demographic data (age, parental education, religion, ethnicity, residence, and occupation), and provider details (skill level, facility type. All data were anonymized before being stored for later analysis (see Appendices).

Sample size and sampling

This was a census-based study of all eligible cases over the five years from January 2019 to December 2024. No prior sample size calculation was performed, as the aim was to obtain the full set of cases that meet the eligibility criteria.

Data analysis/statistical methods

Descriptive statistics (means, medians, frequencies, proportions, percentages) were calculated to summarize demographic characteristics. This constituted the sociodemographic analysis. Bivariate analyses to test for associations, multivariate and geospatial distribution analyses were conducted to explore trends in diagnosis and outcomes, and identify predictors of circumcision complications, given an individual. Logistic regression for model building, followed by internal and external validation, and post-usability testing, was done. Results were presented in tables, graphs, and distribution maps to aid interpretation. All analyses were conducted using Stata version 17. A 5% level of significance was used.

Technical information

Model Development

Identified risk factors were analyzed using logistic regression in Stata (version 17). Significant predictors (p<0.05) were included in the final model, as well as other variables that improved discrimination. Internal and external validation of the model was conducted. The derived formula was programmed into the mobile app for real-time risk calculation. The original data for the study (186 participants) was used for model development and internal validation. External validation was conducted on a different, external dataset of 31 participants.

Mobile Application Development

The app was coded in R Shiny and deployed for Android devices. It outputs categorical risk stratification (low, moderate, and high) and provides basic preventive recommendations tailored to the user's input data.

Pilot Testing

Thirty users (15 healthcare providers, 15 non-healthcare workers) participated in usability testing. Diagnostic accuracy metrics [sensitivity, specificity, positive predictive value (PPV), negative predictive value (NPV), ROC analysis] were calculated.

Ethical approval

This study was reviewed and approved by the University of Health and Allied Sciences Research Ethics Committee (UHAS-REC) under the protocol number UHAS-REC 2023/074. Before data collection, written informed consent was obtained from the guardians of all participating children, ensuring that they fully understood the purpose, procedures, potential risks, and benefits of the study (except for the part of the study that used retrospective data from hospital electronic records). All research activities were conducted in strict compliance with the ethical principles outlined in the Declaration of Helsinki regarding research involving human participants.

## Results

Participant characteristics

Among 186 participants in the primary (model development) study, the mean age was 10 months (SD: 11 months, range: <1 month to 28 years), with the modal age group being infants. Circumcisions were evenly distributed between medical and non-medical/traditional procedures. The mean age for the external validation data (n = 31) was 3.37 years (SD: 5.33, range: <1 month to nine years), with a modal age of one month. The median age for the adult population participating in the usability testing (n = 30) was 25 years.

Descriptive statistics

**Figure 2:**
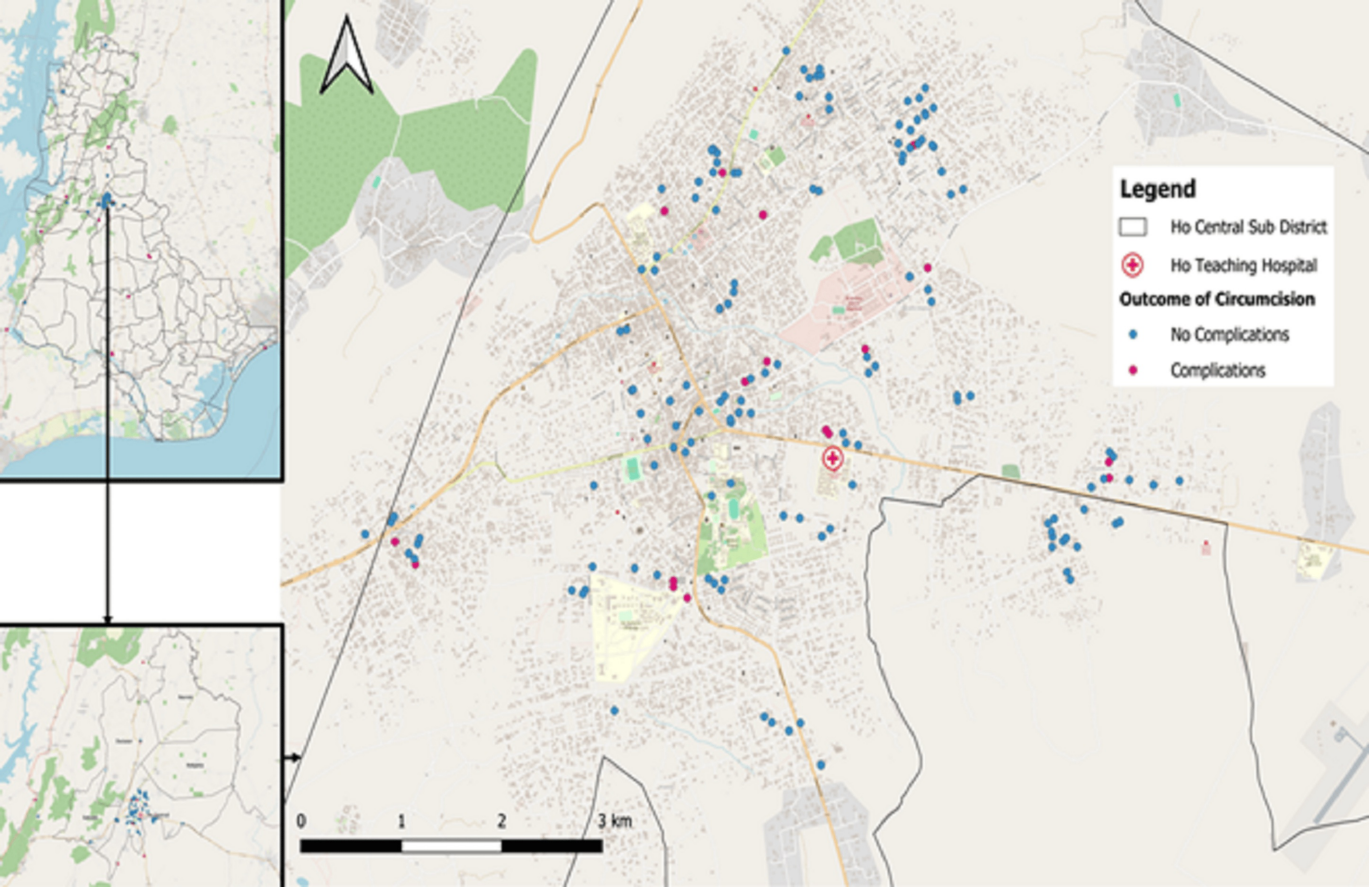
Geospatial distribution map for circumcision cases and circumcision-related complications over the five-year study period The map illustrates spatial clustering and communal patterns of both primary circumcision procedures and reported complications of circumcisions across the study area

A summary of the sociodemographic features of the analyzed population for the primary study is provided in Table 1 [19]. The geospatial distribution of the circumcision cases in this study (normal versus complicated cases) is shown in Figure 2.

**Table 1:** Descriptive and summary statistics of the demographic characteristics of the participants in the study SD: standard deviation

Variable	Category	Value
Age of clients, category 1	≤1 month	28.5%
Age of clients, category 2	1–12 months	41.4%
Age of clients, category 3	2–4 years	14.0%
Age of clients, category 3	5–11 years	9.1%
Age of clients, category 4	≥12 years	7.0%
Age stats 1	Min	1 week
Age stats 2	Max	28 years
Age stats 3	Mean	11 months
Age stats 3	SD	10 months
Occupation of parents category 1	Healthcare	15.1%
Occupation of parents category 2	Education	18.3%
Occupation of parents category 3	Admin	8.1%
Occupation of parents category 4	Creative	11.3%
Occupation of parents category 5	Service/manual	47.3%
Education of parents 1	No formal	2.7%
Education of parents 2	Primary	8.6%
Education of parents 3	Secondary	52.2%
Education of parents 4	Tertiary	36.6%
Religion of parents category 1	Christian	68.8%
Religion of parents category 2	Muslim	26.3%
Religion of parents category 3	Traditionalist	4.8%
Ethnicity of parents 1	Ewe	82.8%
Ethnicity of parents 2	Akan	7.5%
Ethnicity of parents 3	Ga/Adangme/Krobo	3.8%
Ethnicity of parents 4	Northern Tribes	2.7%
Ethnicity of parents 5	Ibo	4.3%
Nationality 1	Ghanaian	95.7%
Nationality 2	Nigerian	4.3%
Place of residence 1	Ho/surroundings	69.9%
Place of residence 2	Adaklu/nearby	8.6%
Place of residence 3	Other towns	21.5%

Bivariate analysis

Circumcision-Seeking Behavior of Participants

Chi-square analysis revealed that multiple sociodemographic characteristics were significantly associated with the choice of circumcision provider and facility. For facility selection, significant associations were found with occupation (χ² = 40.208, p = 0.0000), ethnicity (χ² = 39.022, p = 0.0000), and place of residence (χ² = 16.690, p = 0.0105). Age, education, and religion did not show significant influence on facility choice (p>0.05). In contrast, practitioner choice was influenced by nearly all examined sociodemographic factors, with age (χ² = 29.077, p = 0.0038), occupation (χ² = 25.617, p = 0.0024), education (χ² = 19.612, p = 0.0205), religion (χ² = 18.623, p = 0.0003), and ethnicity (χ² = 33.703, p = 0.0001) showing significant associations. Residence, however, was not statistically significant (p = 0.2734). These results highlight the critical influence of sociodemographic context on circumcision-seeking behavior and provider preference.

Odds ratios comparing the likelihood of choosing a traditional circumciser versus a clinical one, based on participants’ sociodemographic characteristics, gave further insights. Among age groups, individuals in the youngest category ( ≤1 month, neonates) had lower odds of being taken to a traditional circumciser compared to older children. For example, infants had 1.56 times the odds (95% CI: 0.87-2.78) of seeing a traditional provider compared to neonates, p = 0.078. Similarly, education level showed a trend: those with primary or no formal education had increased odds of traditional provider use, with an odds ratio of 2.11 (95% CI: 1.01-4.43), p = 0.047, indicating statistical significance. In terms of residence, individuals from rural areas were more likely to use traditional circumcisers: OR: 2.85 (95% CI: 1.45-5.61), p = 0.002. This reinforces the role of geographic and educational access in shaping health-seeking behavior related to circumcision services.

Predictors of Circumcision Mishaps/Complications Among Participants

Based on the test of associations, the significant risk factors for complications during circumcision included provider skill level (p<0.001), non-clinical facility type (p = 0.015), parental education, parental occupation, residence, and child’s age. Parental religion and ethnicity were not statistically significant predictors, but improved discrimination of the overall model during the logistic regression analysis below (Tables 2, 3).

**Table 2:** Definition of equation variables and the coding guide

Term/variable	Meaning	Coding frame/guide
Coded circumcision complication category	CCD	0 = No complication
Coded age category of the child or patient	AGE_CD	1 = Up to 4 weeks
Coded circumcision complication category	AGE_CD	2 = 4 weeks to 1 year
Coded age category of the child or patient	AGE_CD	3 = >1 year to 5 years
Coded circumcision complication category	AGE_CD	4 = >5 to 10 years
Coded age category of the child or patient	AGE_CD	5 = >10 to 18 years
Coded circumcision complication category	AGE_CD	6 = 18 years and beyond
Coded age category of the child or patient	OCC_CD	0 = Unemployed
Coded circumcision complication category	OCC_CD	1 = Health professional
Coded age category of the child or patient	OCC_CD	2 = Education (lecturers, teachers)
Coded circumcision complication category	OCC_CD	3 = Clerical/managerial/secretarial
Coded age category of the child or patient	OCC_CD	4 = Agriculturist/artisan/vocational/housewife/trader
Coded occupational category of the parent/guardian	OCC_CD	5 = Forces/police/military/pastors
Coded occupational category of the parent/guardian	OCC_CD	6 = Students
Coded occupational category of the parent/guardian	ED_CD	0 = None
Coded occupational category of the parent/guardian	ED_CD	1 = Primary
Coded occupational category of the parent/guardian	ED_CD	2 = Secondary
Coded occupational category of the parent/guardian	ED_CD	3 = Tertiary
Coded occupational category of the parent/guardian	ED_CD	4 = Islamic education
Coded educational level of the parent/guardian	REL_CD	1 = Christianity
Coded educational level of the parent/guardian	REL_CD	2 = Islam
Coded educational level of the parent/guardian	REL_CD	3 = Traditional
Coded educational level of the parent/guardian	REL_CD	4 = No religion
Coded educational level of the parent/guardian	ETH_CD	1 = Ga
Coded religious affiliation	ETH_CD	2 = Ewe
Coded religious affiliation	ETH_CD	3 = Akan
Coded religious affiliation	ETH_CD	4 = Northern tribes + Hausa
Coded religious affiliation	ETH_CD	5 = Igbo, Nigerian/foreign
Coded ethnicity	RES_CD	1 = Rural
Coded ethnicity	RES_CD	2 = Peri-urban
Coded ethnicity	RES_CD	3 = Urban
Coded ethnicity	FAC_CD	1 = Home circumcision
Coded ethnicity	FAC_CD	2 = CHPS/health center/clinic
Coded residential setting (urban/rural)	FAC_CD	3 = District hospital/private hospital
Coded residential setting (urban/rural)	FAC_CD	4 = Specialist private facility/polyclinic/university hospital
Coded residential setting (urban/rural)	FAC_CD	5 = Teaching hospital
Coded facility type where circumcision was done	Practitioner_Type	1 = Doctor
Coded facility type where circumcision was done	Practitioner_Type	2 = Nurse
Coded facility type where circumcision was done	Practitioner_Type	3 = Midwife
Coded facility type where circumcision was done	Practitioner_Type	4 = Herbalist/Wanzam/traditional circumciser

**Table 3:** Chi-square associations between sociodemographic variables and circumcision complication risk

Factor/characteristic	χ² (chi-square statistic)	P-value	Interpretation
Age of circumcision clients	48.999	0	Strong and significant association with age
Parental education level	10.88	0.012	Significant association between education and complication risk
Parental occupation	44.72	0.013	Significant association between occupation and complication risk
Residence (urban vs. peri-urban vs. rural)	29.76	<0.0001	Significant association between residence and complication risk
Ethnicity	5.35	0.253	No significant association
Religion	1.6	0.448	No significant association
Circumciser	26.04	0	Significant association
Circumcising facility	18.51	0.047	Significant association

Multivariate analysis

After backward stepwise multivariate logistic regression analysis (Tables 2, 4) on the primary study data, the logistic regression model equation was obtained with the variables and the derived coefficients, as shown in Table 4.

**Table 4:** Adjusted odds ratios of predictors of circumcision complications; and the respective model coefficients

Variable	Odds ratio	Coefficient (ln(OR))	Std. error	P-value
AGE_CD	3.997	1.386	1.4047	0
OCC_CD	0.98	-0.02	0.2047	0.925
ED_CD	0.768	-0.263	0.2467	0.411
REL_CD	0.956	-0.045	0.7362	0.953
ETH_CD	1.081	0.078	0.2979	0.778
RES_CD	0.433	-0.839	0.1225	0.003
FAC_CD	0.782	-0.245	0.1332	0.149
Practitioner_Type	0.946	-0.056	0.305	0.864
_cons	1.1	0.095	2.152	0.961

The model equation is provided below:

$\log(odds_{CCD}) = 1.0999 + 1.3866 \times AGE_{CD} - 0.0197 \times OCC_{CD} - 0.2643 \times ED_{CD} -0.0451 \times REL_{CD} + 0.0777 \times ETH_{CD} - 0.8362 \times RES_{CD} - 0.2466 \times FAC_{CD} - 0.055 \times \textit{PractitionerType}$

The variables in the equation are defined in Table 2, which also provides the coding frame used in the initial data analysis, logistic regression modelling, validation, and app-building exercises.

Model Performance

**Figure 3:**
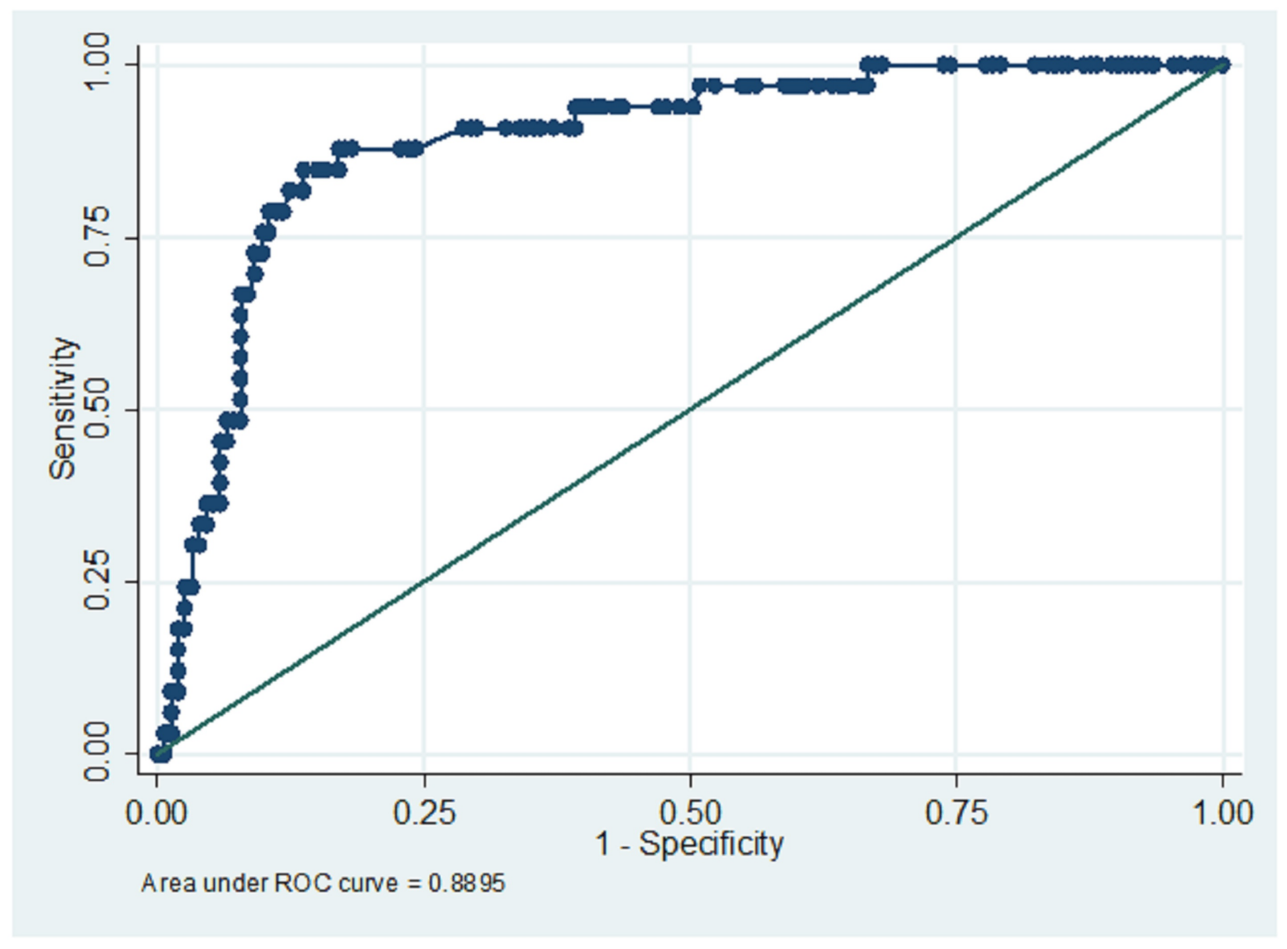
Receiver-operator characteristic curve for the model equation (AUC = 0.78) Receiver operating characteristic (ROC) curve illustrating the performance of the predictive model in distinguishing between circumcision cases with and without complications. The area under the curve (AUC) is 0.78, indicating good discriminative ability of the model. The curve demonstrates the trade-off between sensitivity and specificity across various threshold values. An AUC of 0.78 suggests that the model has a 78% chance of correctly differentiating a randomly chosen complication case from a non-complication case. This evaluation supports the model's clinical utility in risk stratification and decision-making

**Figure 4:**
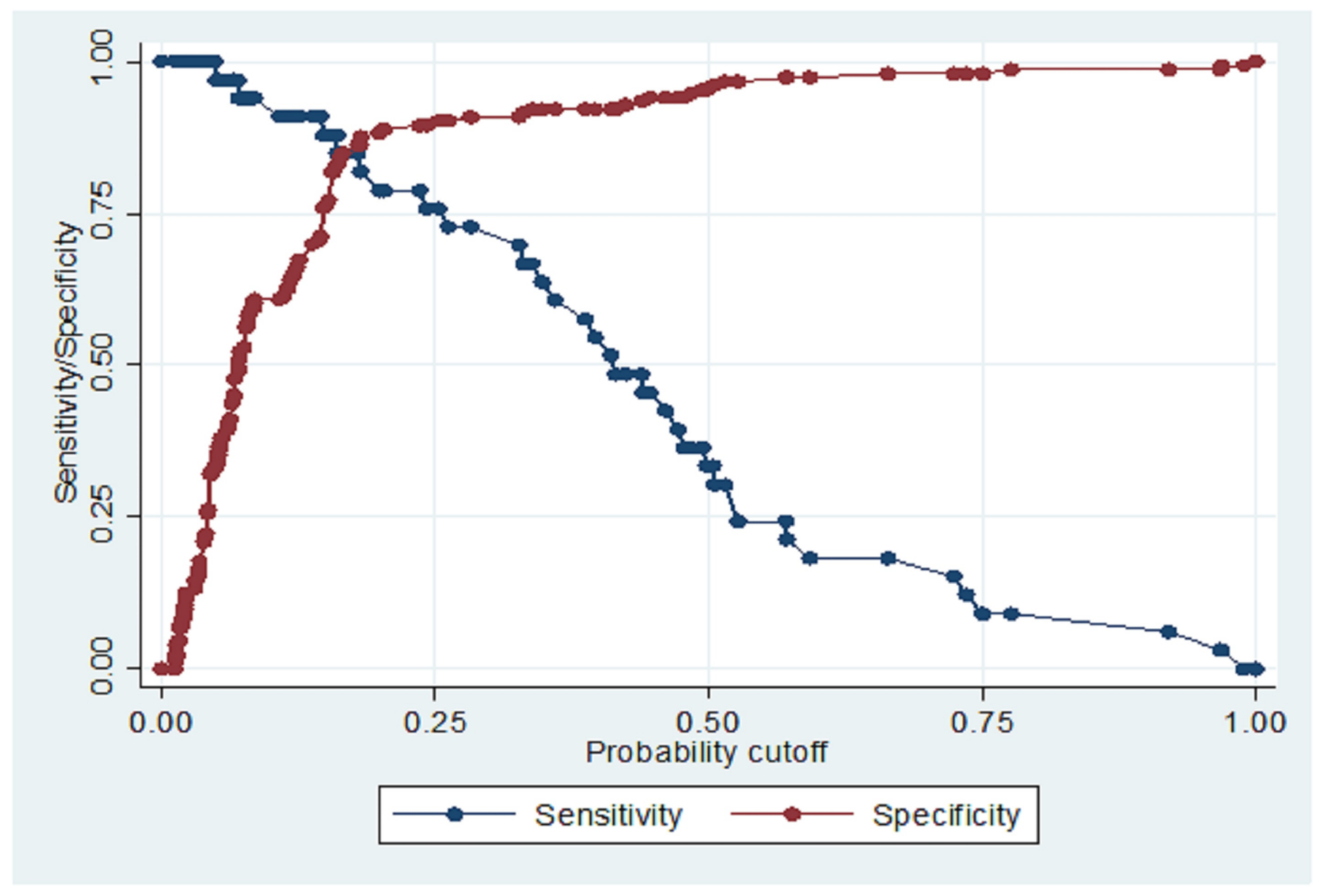
A sensitivity-specificity plot showing the Youden’s index cut-off point (decision point) = 0.200 Sensitivity-specificity plot illustrating the diagnostic performance of the predictive model across varying threshold values. The optimal decision threshold, determined using Youden’s index, is marked at 0.200. This cut-off point maximizes the combined sensitivity and specificity, thereby identifying the most effective point for classifying circumcision-related complications. The plot supports the evaluation of trade-offs between false positives and false negatives in clinical decision-making

**Figure 5:**
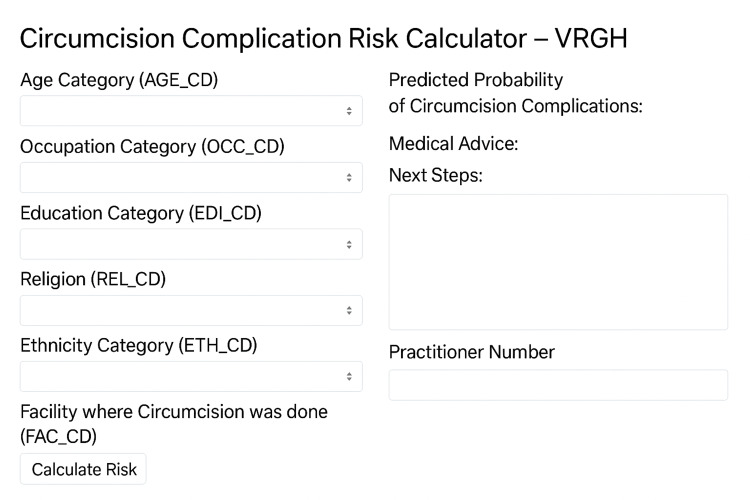
The appearance of the interface of the digital mobile/web application User interface of the digital mobile/web-based application developed for predicting the risk of circumcision-related complications. The interface features structured input fields for key predictors such as age, ethnicity, residence, parental occupation, and facility type. It displays real-time risk estimation outputs and decision-support messages to guide clinical or parental decision-making. Designed for usability across devices, the interface supports both mobile and desktop platforms to ensure accessibility for healthcare providers in diverse settings

The internal validation and external validation results, and their confusion matrix tables, are shown in Tables 5-6 and Figures 3-4. The mobile app categorized patients into low, moderate, and high risk based on demographics and provider-related factors, including circumciser skill and facility type. The model achieved a specificity of 96.08%, a PPV of 64.71%, and an NPV of 86.98%, correctly classifying 84.95% of cases. It, however, had a modest sensitivity of 33.33%. The Hosmer-Lemeshow test (chi² = 11.05, p = 0.199) indicated good model fit, and the ROC curve (AUC = 0.7895) suggested excellent predictive performance. There was no overfitting and multicollinearity (Table 5). Youden's Index Decision point was 0.200. (Tables 5, 6; and Figures 3, 4, 5).

**Table 5:** Internal and external validation results table: circumcision complication risk model AUC: area under the receiver operating characteristic curve; VIF: variance inflation factor

Metric	Internal validation	External validation
Shrinkage factor	0.92 (heuristic shrinkage)	N/A
AUC (C-statistic)	0.78 (after bootstrap correction)	0.976 (from confusion matrix and predicted probabilities)
Calibration-in-the-large	≈ 0 (no systematic over/underprediction)	Visual alignment observed using a risk threshold of 0.20
Calibration slope	≈ 0.92 (matches shrinkage factor)	Implicitly good (24/24 true positives at 0.20 threshold)
Hosmer-Lemeshow test	p > 0.05 (good fit)	Not applicable
Discrimination	Acceptable (AUC 0.78)	Excellent (AUC 0.976)
Multicollinearity (VIF)	All VIFs < 2 (no multicollinearity)	Not assessed separately
Overfitting evidence	Low optimism (Δ AUC = 0.03)	No overfitting observed (model generalized well)
Risk threshold used	0.20	0.20
Accuracy	84.95%	(24+6)/31 = 96.8%
Sensitivity (recall)	33.33% (increased to 70% with moderate risk cut-off 0.200–0.295)	24 / 24 = 100%
Specificity	96.08%	6 / 7 = 85.7%
Precision (PPV)	64.71%	24 / 25 = 96.0%
F1 Score	44.4%	2 × (96.0 × 100) / (96.0 + 100) = 97.9%
False positive rate (FPR)	3.93%	1 / 7 = 14.3%
False negative rate (FNR)	66.7%	0 / 24 = 0%
True positive rate (TPR)	33.3%	100%
True negative rate (TNR)	96.07%	85.7%

**Table 6:** Confusion matrix tables for internal and external validation

Validation set	Prediction	Actual CCD = yes	Actual CCD = no	Total
Internal validation	Predicted = yes	8	6	14
Internal validation	Predicted = no	15	157	172
Internal validation	Total	23	163	186
External validation	Predicted = yes	24	1	25
External validation	Predicted = no	0	6	6
External validation	Total	24	7	31

Mobile App Usability Testing

User feedback revealed high (>80% of responders) satisfaction with app simplicity, clarity of results, and decision-support features. Providers particularly appreciated the immediate risk categorizations to guide parental counseling (Table 7; figure in the Appendices section).

**Table 7:** App usability testing with post-usability survey questionnaire

Usability aspect	N (%) (N=30)
Satisfactory to use	21 (70%)
Easy to use	24 (80%)
Simple and pleasant interface	24 (80%)

## Discussion

The findings of the analyses in this study underscore the substantial influence of sociodemographic factors on circumcision-seeking behavior among participants. Significant associations were observed between occupation, ethnicity, and residence and the choice of facility, indicating that structural and cultural access issues shape where caregivers seek circumcision services. Conversely, age, education, and religion showed no significant effect on facility choice (p>0.05), suggesting that institutional access may transcend individual beliefs when facility-level decisions are made. However, nearly all sociodemographic characteristics significantly influenced the choice of circumciser, with age (p = 0.0038), occupation (p = 0.0024), education (p = 0.0205), religion (p = 0.0003), and ethnicity (p = 0.0001) showing strong associations. These results reflect deep-rooted cultural, educational, and geographic patterns in provider preferences-factors extensively documented in similar studies from Kenya, Nigeria, and Uganda [3-5].

Odds ratios further illuminated these dynamics. For instance, younger children (≤1 month) had lower odds of being circumcised by traditional providers, likely reflecting a preference for clinical safety in neonatal care, as echoed in systematic reviews highlighting reduced risk when circumcision is performed early and clinically [1,2]. Infants had 1.56 times the odds (95% CI: 0.87-2.78) of using traditional providers compared to neonates (p = 0.078), and participants with primary or no education had 2.11 times higher odds of utilizing traditional circumcisers (95% CI: 1.01-4.43, p = 0.047), reinforcing the role of educational empowerment in shaping safer health choices [5,6]. Geographic disparities were also evident, with rural residents showing 2.85 times the odds of consulting traditional circumcisers (95% CI: 1.45-5.61, p = 0.002), which aligns with findings related to rural underutilization of skilled care and infrastructure limitations [4,6]. This supports calls for mHealth and decentralized interventions to bridge care gaps in hard-to-reach communities [7,9,16-18].

Together, these findings suggest that all the above factors could ultimately predict circumcision outcomes. They also highlight the need for targeted education, community-based interventions, and policy-level reforms that consider the role of sociodemographic characteristics on health-seeking behavior in male circumcision. It may also lend credence to the proposition that mHealth tools -such as those piloted in other surgical and preventive care models - could personalize circumcision risk communication and redirect high-risk profiles toward safer clinical pathways [11,12]. This would ultimately reduce adverse outcomes, as also demonstrated in related studies [1,6]. In line with the foregoing, this study also successfully developed and validated a logistic regression model and converted it into a digital circumcision risk calculator. This tool demonstrated strong model performance and favorable user feedback. By leveraging sociodemographic, spatial distributional, implicit health-seeking behavioral factors, and procedural biomarkers, the app allows evidence-based risk stratification: an innovation not previously documented in Ghanaian circumcision practices [11-20].

The model’s emphasis on specificity was deliberate, aiming to prioritize precision in identifying truly high-risk individuals rather than over-labeling. The app’s lower sensitivity is consistent with its purpose as a preventive screening tool rather than a diagnostic one, where specificity and reliability in risk identification are more critical [14,15]. Our focus ensures fewer false positives, which could otherwise create unnecessary anxiety among parents and providers. The app is accessible at https://wanzam-frontend-v2.vercel.app/ (included in the references [20]).

On internal validation, the circumcision complication risk model demonstrated acceptable performance, with an AUC of 0.78, indicating good discriminatory power within the development dataset. The calibration slope of approximately 0.92, closely matching the heuristic shrinkage factor, confirms the model’s robustness and minimal overfitting (ΔAUC = 0.03). The Hosmer-Lemeshow goodness-of-fit test (χ² = 11.05; p = 0.199) supports adequate calibration. Multicollinearity was ruled out with all variance inflation factors (VIFs) <2, confirming the stability of predictors used in the model. Although the sensitivity was modest at 33.3%, this was offset by a specificity of 96.08%, reflecting a conservative classification strategy favoring high precision (PPV: 64.1%) and low false positive rate (FPR: 3.93%). The F1 score of 44.4% highlights a trade-off between recall and precision, which is acceptable given the model’s design as a risk stratification tool to guide resource prioritization and preventive strategies in circumcision care [1,3,4,6].

External validation showed superior performance, with an AUC of 0.976 and accuracy of 96.8%, confirming the model’s generalizability across different user groups and clinical settings. Importantly, the external cohort achieved 100% sensitivity and 96.0% PPV, correctly identifying all true complications at the selected risk threshold of 0.20, with only one false positive. This demonstrates excellent calibration-in-the-large and strong alignment between predicted probabilities and observed outcomes. Usability testing using the PUSQ revealed that 70% of users found the app satisfactory, and 80% rated it easy to use with a simple and pleasant interface, aligning with findings from other mHealth implementation studies [7-9,11,12,16-18]. These results affirm the app’s operational feasibility and reinforce its role as an implementation research tool for improving circumcision safety in Ghana, especially in low-resource, high-burden settings [5,6,14,17]. Our findings align with prior studies advocating technology-assisted medical decision-making in low-resource settings [16-20]. Future research should aim to expand datasets and conduct extensive prospective external validation studies to enhance generalizability, integrate machine learning algorithms to boost predictive accuracy, and incorporate real-time feedback loops for continuous app improvement.

The mobile app-based digital risk calculator for circumcision complications showed favorable internal and external validation results. Its post-usability testing outcomes were also satisfactory. It could significantly contribute to safer circumcision practices in Ghana. By providing real-time, personalized risk assessments, it empowers healthcare providers and parents, potentially reducing adverse outcomes. Ongoing improvements will focus on scaling and refining the tool across diverse populations. In light of these findings, the digital risk calculator should be considered an important adjunct in national efforts to improve circumcision safety, especially in settings with limited access to skilled personnel or standardized protocols. We recommend that healthcare policymakers in Ghana and similar low-resource settings formally integrate the app into child health and surgical safety programs. This could be done through the Ghana Health Service's digital health strategy, with tailored training modules for frontline workers. Professional bodies and regulatory authorities, such as the Medical and Dental Council and the Nurses and Midwives Council, adopt the tool as part of continuing medical education and quality assurance processes, especially for providers involved in male circumcision services. Community health education programs utilize the app to engage and inform parents and caregivers about potential risks, thereby supporting informed decision-making and early referral of high-risk cases.

Urologic surgeons in Ghana could consider adopting this tool as a viable decision support tool in the area of preventing and controlling circumcision mishaps in Ghana. For the field of Public Health and Global Health, this tool is a potentially useful tool for implementation research, as a practical approach to stemming the tide of the kind of rueful circumcision mishaps that urologists and Pediatric surgeons in Ghana and Sub-Saharan Africa are regularly confronted with. App developers and implementation scientists could work collaboratively to ensure language localization, user interface enhancements, and offline usability to accommodate diverse populations, including rural and underserved areas. Researchers and academic institutions further evaluate the app’s long-term impact on circumcision outcomes, complication rates, and parental satisfaction across different healthcare settings. Ultimately, this digital innovation demonstrates that simple, evidence-based tools can bridge gaps in surgical safety and patient counseling. Scaling up this approach could significantly reduce preventable injuries and complications from circumcision, while promoting digital transformation in pediatric surgical care in sub-Saharan Africa and beyond.

Limitations

Limitations include the small sample size for external validation and the unavailability of data on important procedural factors, including the method of circumcision and the use of anesthesia or otherwise. Data on parental age and previous circumcision experiences (which may influence health-seeking choices) were also not available.

## Conclusions

Circumcision-related complications remain a significant public health concern in Ghana, especially from non-clinical and unregulated providers. Risk factors such as patient age, circumciser skill, and procedure setting influence complication rates. There are currently no validated, context-specific digital tools in Ghana for predicting and preventing circumcision-related complications. This study presents the first internally and externally validated risk calculator for circumcision complications in Ghana. It integrates sociodemographic and procedural variables into a mobile application for real-time, pre-procedure risk profiling. The tool offers a practical, mHealth-based solution to support healthcare workers and parents in making safer circumcision decisions.

## Appendices

**Table 8:** Data collection tool for the study

Section	Parameter
Demography	Hospital ID No.
Demography	Age
Demography	Occupation of Parents
Demography	Educational Level of Parents
Demography	Religion
Demography	Ethnic Group
Demography	Nationality
Demography	Residence
Clinical parameters	Full Blood Count
Clinical parameters	For referred circumcision disasters or complicated circumcisions, also record the following (same applies for phimosis and paraphimosis)
Clinical parameters	Note: For normal circumcisions done in Trafalgar, record all the above parameters
Clinical parameters	Presentation (Acute Emergency/Cold Presentation at OPD)
Clinical parameters	Presenting Complaints
Clinical parameters	Duration of Symptoms
Clinical parameters	Town where Circumcision was Done
Clinical parameters	Facility where Circumcision was Done
Clinical parameters	Who Made the Referral Decision? (Relatives or Circumciser)
Clinical parameters	Practitioner Type (e.g., Wanzam, Herbalist, Pastor, Midwife, Nurse, Grandparent, MO, PA, Others - Specify)
Clinical parameters	Associated Diagnoses (e.g., Epispadias, Hypospadias, Chordee, Hooded Prepuce, Hydrocele, Testicular Mass, Undescended Testes, Meatal Stenosis)
Clinical parameters	Examination Findings (Detailed)
Clinical parameters	Exact Type of Circumcision Complication (e.g., Glans or Penile Amputation, Urethrocutaneous Fistula, Incomplete Circumcision, Cicatrization, Chordee - Specify)
Clinical parameters	Type of Surgical Repair/Intervention
Clinical parameters	Senior Most Attending/Operating Surgeon (HO/SHO/MO/Resident/Specialist/Consultant)
Clinical parameters	Anesthesia Used (Local, Spinal, General, Others)
Clinical parameters	Outcome of Repair
Clinical parameters	Complications
Clinical parameters	Follow-Up
Clinical parameters	Recurrence/No Recurrence
Clinical parameters	Additional Comments
